# Etiopathogenesis of type 1 diabetes mellitus: prognostic factors for the evolution of residual β cell function

**DOI:** 10.1186/1758-5996-1-25

**Published:** 2009-12-04

**Authors:** Sergio A Dib, Marilia B Gomes

**Affiliations:** 1 Endocrinology Division, Department of Medicine of Federal University of São Paulo, SP, Brazil; 2 Diabetes Division, Department of Medicine of State University of Rio de Janeiro, GB, Brazil

## Abstract

Type 1A diabetes mellitus (T1ADM) is a progressive autoimmune disease mediated by T lymphocytes with destruction of beta cells. Up to now, we do not have precise methods to assess the beta cell mass, "*in vivo*" or "*ex-vivo*". The studies about its genetic susceptibility show strong association with class II antigens of the HLA system (particularly DQ). Others genetics associations are weaker and depend on the population studied. A combination of precipitating events may occur at the beginning of the disease. There is a silent loss of immune-mediated beta cells mass which velocity has an inverse relation with the age, but it is influenced by genetic and metabolic factors. We can predict the development of the disease primarily through the determination of four biochemically islet auto antibodies against antigens like insulin, GAD65, IA2 and Znt8. Beta cell destruction is chronically progressive but at clinical diagnosis of the disease a reserve of these cells still functioning. The goal of secondary disease prevention is halt the autoimmune attack on beta cells by redirecting or dampening the immune system. It is remains one of the foremost therapeutic goals in the T1ADM. Glycemic intensive control and immunotherapeutic agents may preserve beta-cell function in newly diagnosed patients with T1ADM. It may be assessed through C-peptide values, which are important for glycemic stability and for the prevention of chronic complications of this disease. This article will summarize the etiopathogenesis mechanisms of this disease and the factors can influence on residual C-peptide and the strategies to it preservation.

## 

We are presently going through a revaluation of the knowledge acquired in the last decades regarding the etiopathogenesis of type 1 diabetes mellitus (T1DM).

## Type 1 diabetes classification

Nowadays, we may subdivide T1DM in three groups from the etiological point of view: autoimmune, idiopathic and double. The autoimmune group is represented by: type 1A, which is polygenic and it is the most frequent type of this disease, corresponding to approximately 80-90% of all T1DM cases [1]. The other subtype of this group is the latent autoimmune diabetes in adults (LADA)[2], which appears after the age of 35 and is frequently associated with other autoimmune endocrine diseases. The third subtype includes called "monogenic" T1DM. They correspond to T1DM of the autoimmune polyglandular syndrome type 1A [3] and of IPEX syndrome (Immune Dysfunction, Polyendocrinopathy, Enteropathy, X-linked) [4].

Type 1B, also called idiopathic, has all the clinical features of type 1A, but the autoimmune component is not detected [5]. Another 1B subtype is the fulminant diabetes most described in Asian peoples, mainly Japan, China and Korea, characterized by a short clinical history, before to the first acute metabolic decompensation, presents the impairment of beta and alpha cells of the pancreatic islet and no autoimmune etiology [6].

Finally, the denomination of mixed, 1.5 or double (type 1 plus type 2) diabetes has been proposed when we have the type 1A (autoimmunity) plus type 2 (obesity, insulin resistance, dyslipidemia) diabetes characteristics in the same individual [7].

In this review, we will cover only autoimmune type 1A diabetes.

## Type 1 A diabetes

Type 1 A diabetes is a chronic inflammatory disease which leads to selective destruction of beta cells in pancreatic islets [8]. Such cells are affected by a process involving specific cellular and humoral autoimmunity mechanisms against their antigens. A failure of regulatory T cells (T- regs) on this process is involved [9]. However, even though type 1 A diabetes is one of the most widely studied autoimmune diseases over the last decades, its complete natural history has not been fully clarified yet.

One of the subtypes of type 1A diabetes is the "monogenic" diabetes, associated with the Autoimmune Polyglandular Syndrome Type 1 [10]. Such syndrome, also as APECED (Autoimmune Polyendocrinopathy- Candidiasis-Ectodermal Dystrophy) is more frequent in children and pre-puberal individuals and is associated with the mutation of the autoimmune regulatory gene (AIRE), located in chromosome 21, that it is important to the regulation of autoimmune mechanisms [3]. A change in this gen results in autoimmune reactions to different antigens expressed in peripheral tissues due to a failure in regulating the presentation of such antigens in the thymus for its recognition as "self-antigens". As it is already known, a large part of autoimmune regulation occurs at the thymus level, so that when peripheral lymphocytes go through the thymus, they receive information about antigens to which they must react or not. When the individual suffers change in the AIRE gene, the selection criteria fail and these patients start to react against antigens to which they should not react. Type 1 diabetes is observed in 13-20% of these patients. They are generally referred by other specialists, because the first clinical manifestation, most of the times, is not hyperglycemia. The most frequent manifestations of this syndrome are mucocutaneous candidiasis, Addison's Disease and hypoparathyroidism [3]. It is interesting that in such patients, the glutamic acid decarboxylase antibodies (GADA), that in classical type 1A diabetes indicate pancreatic β cell lesion, are more related to the presence of enteropathy [3].

The second "monogenic" subtype of type 1 A diabetes is a rare type of diabetes which occurs in children and is associated with changes in genes located in chromosome X [11]. In this type of diabetes, children present mutation in the gene which codifies the express of FOX-P3 in CD4+ and CD25+ cells. Such changes lead to a disability of generating regulatory T cells and the development of early autoimmunity against several organs. Thus, it is characterized by the onset of a different type of diabetes, dependent on exogenous insulin, enteropathy, growth deficiency and early death in such children [4].

Nevertheless, the polygenic group type 1 A diabetes is the most frequent form of T1DM and it is accountable for approximately 90% of these cases.

## Genetic Predisposition

Type 1 A diabetes is considered by some authors a polygenic and by others, an oligogenic, but heterogeneous disease. To date, several loci in different chromosomes are related to the genetic susceptibility of this type of diabetes [12]. Such locis are denominated IDDM1, IDDM2, IIDM3, etc. Many of these loci are also related to the predisposition to other autoimmune diseases, such as multiple sclerosis, celiac disease, ankylosing spondylitis and Hashimoto's thyroiditis.

The most important genes are located inside the major complex of histocompatibilty (MHC) in the region of class II of the HLA system, particularly molecules DR, DQ and DP in chromosome 6p21.31 [13]. These are called IDDM1 and are responsible for about 45% of the genetic susceptibility of type 1 A diabetes. Most of these data come from Caucasian populations from Europe and North-America. In these studies, approximately 95% of patients have class II antigens HLADR3 or - DR4 and 55 - 60% of them are heterozygotes DR3/DR4. Genotype DR3/DR4 offers higher risk for type 1 A diabetes, with a synergic mode of action, followed by homozygote DR4 or DR3, respectively.

The data suggest that DR4 may offer this susceptibility in a dominant manner, while DR3 as a recessive feature. The latest studies using molecular biology techniques have demonstrated that the locus HLA-DQ is more narrowly associated with the susceptibility to type 1 A diabetes. Such locus codifies important proteins at the presentation and recognition of antigens by the immune system. In Caucasians, the heterodimers HLA-DQ (alpha chains denominated DQA1 and beta chains, DQB1) codified by the alleles DQA1*0301, DQB1*0302 and DQA1*0501, DQB1*0201 have the strongest association with type 1 A diabetes and are respectively not balanced at the connection to alleles HLADR4 and DR3. On the other hand, among four common DR2 haplotypes, observed in Caucasians, DQA1*0102, DQB1*0602, DRB1*1501 are negatively associated with type 1 A diabetes and are related in less than 1% of the majority of populations studied, including Asians, Afro-Americans and Mexican-Americans. This protection seems to have a dominant effect, as the presence of DQB1*0602 protects from diabetes, with rare exceptions, even in the concomitance of alleles of the HLA system of high risk of the disease. In other ethnical groups, the susceptibility for type 1 A diabetes associated with the genes of the HLA system may involve other alleles. Particularly in Brazil, the latest studies in populations of the Northeast [14] and Southeast [15] show higher frequency of antigens DRB1*03 and DRB1*04 in type 1 diabetes than in normal controls. One of the genes related to the protection is DRB1*11 in both Brazilian populations studied [14,15].

For nearly 20 years, Bell et al[16] found out that variations in the number of nucleotide elements repeated (*Variable Number of Tanden Repeats *- VNTR) of the 5' portion of the insulin gene were associated with the development of type 1 A diabetes. A longer group of repetitions was associated with a reduced risk of diabetes. Such studies have been replicated and have demonstrated that the important locus is clearly limited to the insulin gene [17]. It is denominated IDDM2, located in chromosome 11p15.5 and contributes with approximately 10% of this disease susceptibility. One of the mechanisms suggested for the susceptibility and resistance associated with IDDM2 is related to the influence of VNTR in the transcription of insulin in the thymus, necessary to establish self-tolerance during body growth and development.

In a third place of this disease prediction is a lymphocyte specific phosphatase (PTPN22) gene [18].

Another locus associated with a modulation of the immune response and with type 1 A diabetes, in some populations, is IDDM12 in chromosome 2q33, related to a protein 4 of cytotoxic T lymphocytes (CTLA-4)[19].

In summary, IDDM1 and IDDM2 compete to approximately 50% of the family aggregation of type1 A diabetes. The remaining 50% probably occur due to additional inherited polymorphisms/mutations. The lack of 100% concordance in identical twins could be due to somatic mutations, gene rearrangements by chance (e.g., rearrangements in T cell receptors) or to environmental factors.

Ultimately, it is important to emphasize that genes may have unique behavior among autoimmune diseases, where a group is related to susceptibility and another is related to a significant protection and, many times, surpassing susceptibility. Studies using recent techniques for identifying genes and the formation of cooperative study groups must increase the speed of appearance of susceptibility genes for type 1 A diabetes.

## Environmental Factors

The incidence of type 1A diabetes has increased dramatically in many countries during the past four decades; it is likely that some factors in the environment are changing. Several environmental factors have already been associated with the development of type 1 A diabetes, such as viruses, certain constituents of diet and preservatives food.

The gastrointestinal tract is likely the main system through which non-self antigens gain access. Recent data from literature have emphasized the importance of the intestinal barrier, which is an entry door for viruses and proteins. In early childhood, such barrier is immature, which allows the passage of several antigens, and when these antigens are presented to T lymphocytes by the antigen presenting cells (macrophages), they will trigger an immune response which might result in a autoimmune process. Fasano A et al [20,21] have recently reported a novel protein, zonulin, that modulates intestinal permeability by disassembling the intercellular tight junctions. Such protein may be assessed in the plasma and studies demonstrate that its serum concentration is increased in patients with type 1 A diabetes in relation to their relatives and to controls[22], suggesting that a possible link between genetic susceptibility, increased intestinal permeability, environmental exposure to non-self antigens and development of autoimmune disease.

The influence of several environmental factors in the development of type 1 A diabetes, such as birth-related factors (type of birth, new-born weight), infections, vaccinations, diet components and psychosocial factors are being assessed in a longitudinal study with children since their first months of life up to the age of 15 (TEDDY study). People interested in such study may enroll through the electronic address http://Teddy.epi.usf.edu/TEDDY/index.htm.

## Autoimmune Process

The selective destruction of beta cells of pancreatic islets in T1ADM is the result of a complex interrelation among beta cells, the immune system and environmental factors in individuals genetically susceptible. However, the mechanisms which start the changes in this interrelation and lead to the development of T1DM are not yet fully clarified and, up to now, there are no specific genes or proteins for most of the cases of T1ADM.

Proteins are involved in most cellular processes and the cumulative expression of certain proteins may reflect the specific activities of these cells, thus proteomics may be useful to describe the protein profile expression of cells and of their diabetic phenotype. In this sense, the study of proteomics and metabolomics is an area which seeks the development of methods able to characterize, through biologic samples, bioindicators able to diagnose diseases in initial stage, to predict levels of susceptibility and to monitor the disease progression and its response to therapies.

Proteomics has been applied in studies of beta cell differentiation, exposure of islets to cytokines, manipulation of the nutritional pattern of islets and of transplanted cells. Though these studies have revealed a complex and detailed scene of the protein expression profiles of these cells, their functional implications have not been clarified yet. Up to now, data indicate that beta cells participate actively in their self-destruction during the development of T1DM. Likewise, it seems that there is not any isolated protein responsible for the disease development, but there are serial reactions which favor the transition of dynamic stability of healthy beta cells to dynamic instability and eventual destruction of beta cells [23].

Considering the above mentioned, we may infer that the T1DM prevention is difficult. It is supposed that, in the autoimmune process evolution, individuals produce different proteins, antigens with the expression of different epitopes which were not detected initially, contributing to the process perpetuation. According to the suggestion of a recent article[24] in the beginning of the autoimmune process against pancreatic beta cells, we may have three or more antigens, but at the end, there are endless antigens which are activating the process, i.e., the greater the beta cell lesion, the more antigens are expressed, which will reactivate the process. This proposal covers a new concept for the natural history of T1ADM which, in its preclinical stage, would be characterized by a succession of relapses and remissions with interrelation between regulatory T cells (T-regs) and effectors cells, and regeneration of beta cells up to the moment when the percentage of beta cell destruction would no longer allow a proper insulin secretion, resulting in the expression of hyperglycemia. Within this context, it becomes important to mention the low capacity of regeneration/neogenesis of beta cells mainly when they are exposed to hyperglycemia, which is a stimulus metabolic factor to the insulin secretion, but it is also glycotoxic. When proper glycemic control is instituted at the beginning of the disease, these cells have acquiescence and may to keep the levels of C-peptide secretion for a additional period of time.

After presenting the antigen by the macrophages to T lymphocytes, at least four types of answer may be induced in the immune system: Th1 (cellular immune response), Th2 (humoral immune response), Th17 (cellular immune response potentialization) and T-regs (which take the control of immune cellular reactions). Nowadays, T1DM is considered a T- reg disease, both by its decrease or by its function alteration (e.g., T lymphocytes of Th1 response with Th17 which do not obey the regulation of T- reg cells). This was confirmed by the publication of T1DM cases in individuals who did not produce antibodies through congenital agamaglobulinemia [25,26]. In such individuals, the Th2 response was absent and thus there was no antibody production. At the same time, it is also known that during the pathogenetic process, peripancreatic T lymphocytes play an important role in the transmission of local reactions in islets to systemic cells [27].

The anti-islet antibodies circulating also express the inflammatory lesion taking place in the pancreas. In T1ADM the most studies autoantibodies are classical anti-islet (evaluated through the indirect immunofluorescence method and using as substract cry preserved human pancreas sections), glutamic acid decarboxylase antibodies (GADA), anti-tyrosine-phosphatase (IA2/ICA512) antibodies and anti-insulin autoantibodies. The presence of autoimmunity against the pancreatic islets is considered when the individual has one or more antibodies persistent for at least 3 to 6 months. It is important to confirm these antibodies at least two times in three different occasions.

Antigens ICA512 (IA-2) and later IA-2 β (fogrin-phosphatase of insulinoma granules) were isolated independently by different investigators [28-30]. Almost all antibodies which react with IA-2β also react with IA-2, while approximately 10% of patients who develop T1ADM have antibodies reacting against IA2, but not IA-2β. Thus, during the routine, the IA-2β essay is unnecessary.

So far, insulin and pro-insulin are the only specific antigens of beta cells (all the other antigens described can also to be founded at other cells). The insulin molecule epitope recognized by the liquid phase essays seems to be homogeneous [31]. The anti-insulin antibodies react with conformational molecule epitope (not against chains A or B separately) [32]. Nowadays, studies has been shown that this epitope is between the positions 23 and 30 in the insulin beta chain. Essays for anti-insulin antibodies have more specificity for the disease than the ones against proinsulin. Anti-insulin antibody values correlate inversely with the age when T1ADM develops. Thus, values above 2000 nU/ml are almost exclusively present in children who develop this disease before the age of 5 and less than 50% of the individuals who develop T1ADM after the age of 15 years old may have these autoantibodies. Such antibody value is, to some degree, genetically determined and associated with DR4 [33], DQ8 [32], DQA1*0102, 0201,0301, 0401 [33]. There are data suggesting that the anti-insulin antibody value among individuals with positive ICA is inversally related to time of their evolution to the clinical disease [34]. Usually, anti-insulin antibodies are the first to appear in children who develop T1DM [34-36], mainly in children below 1 year of age. From five children with early (around 8 months old) high anti-insulin antibodies titers, 4 developed T1ADM below the age of 3 years old [35].

Among T1ADM relatives children who are persistently positive only to anti-insulin antibodies rarely develop clinical T1DM [33], but a high percentage of those who show another anti-islet cell antibody evolve to T1ADM after 10 years of age. Adolescents and adults present low concentration of anti-insulin antibodies.

The use of vaccines constituted from fragments 9-23 of the insulin B chain, as recently performed with GAD-65, has been tested in experimental T1ADM models to verify their immunomodulate power in the natural history of the disease [37].

The last of antigens described in the autoimmune process against pancreatic beta cells was one of the bivalent cation (zinc) transporters [38]. Such antigen was characterized through the microarray technique and showed specificity of approximately 80% and high clonal pancreatic frequency. When this new antibody were analyzed together the antibodies previously described (GADA, anti-insulin and anti-IA2), it was possible to increase the autoimmune T1DM diagnosis from 80-85% to 98.2%.

The analysis concerning the evolution profiles of individuals with multiple anti-islet autoantibodies was assessed in the DAISY study [39]. In this study where children are assessed consecutively since birth regarding these autoantibodies and glucose tolerance, it was possible to characterize three evolution profiles: 1- Positive transitory (individuals transitorily positive for antibodies who do not develop the disease); 2- Non-diabetics (individuals persistently positive for autoantibodies who do not develop the disease) and 3- Pre-diabetics (individuals persistently positive for multiple autoantibodies who develop the disease).

The beta cell recovery function after the clinical diagnosis of the disease is extremely rare. One report [40], a few years ago, shown a patient (male, 13 years old) with a classical T1DM(low C-peptide secretion, DQB1*0303,0501), that was initially treated with insulin but it was discontinued after eleven months. Four years after de clinical diagnosis the reevaluation of this patient showed an improvement in the C-peptide secretion and normal glucose tolerance. The immunological assessment showed that GADA were initially positive, but the concentration was low (± 10 U/ml), persisting weakly positive (± 2 U/ml) and anti-insulin and IA2 autoantibodies which were initially positive became negative later. At the same time, the lymphocyte response to proinsulin showed elevated concentration of interleukin 10, which is one of the protective interleukins for the autoimmune T1DM process and trophy for T-reg cells, demonstrating the important role of these cells in the process.

However, this patient evolution is an exception, as in most cases there is a progressive decline in C-peptide secretion during the natural history of this disease, following the start of glycemic instability. In our experience, approximately 60% of T1DM with less than 6 months of clinical diagnosis present residual C-peptide secretion (baseline > 0.6 ng/ml) but which presents a significant drop after 2 to 3 three years of diagnosis and only 3% of individuals over 5 years of diagnosis present positive C-peptide secretion.

## Preservation of the residual C-peptide secretion

Today, one of the therapeutic goals in T1DM is the preservation of the residual C-peptide secretion that is detected in a significant percentage of patients at diagnosis and which potentially may influence the clinical course of the disease.

Several studies have been demonstrated that residual C-peptide secretion, after T1ADM diagnosis, depends on genetic factors, the patient's age at the diabetes diagnosis, the number of anti-islet antibodies and the residual C-peptide secretion. At the same way, intensive insulin therapy and immunomodulators drugs may be useful in this direction.

Regarding genetic factors, the first ones studied were from the HLA system. Among these, A24^+^DQA1^+^03^+ ^and DR9^+ ^have been associated to a higher velocity of C-peptide levels decrease. Patients who do not have these genes would keep a better beta cell function and higher C-peptide secretion [41].

Two other genetic factors related to residual C-peptide secretion are PTPN-P22 (*protein tyrosine phosphatase non-receptor type 2*[42] and one of the vitamin D receptor polymorphism (Fok1)[43].

PTPN22 gene codifies a lymphoid specific phosphatase synthesis, known as LYP, which is important to inhibit T lymphocites activation. A change in the LYP function leads to an alteration in regulatory T cells CD4^+^CD25^+^, making the system less powerful to suppress immune response against autoantigens. The residual C-peptide secretion follow-up on the first 12 months of the disease in T1DM patients showed that it decreased in patients who had alterations in such gene [42].

Among unconventional actions of this vitamin is its immunomodulating function [43]. In this way, we assess the relation of the frequency of one of vitamin D polymorphism receptor, FOK-1, in a group of T1DM with 7 years average period of diagnosis and we verified that the patients who had this polymorphism presented lower residual C-peptide secretion [44].

It is classically known that children and adolescents (0 to 17 years old) present at T1DM diagnosis a lower C-peptide response to a mixed meal than adults [45]. However, there are recent literature data which demonstrates that, despite this initially higher C-peptide reserve in adults, the dropping speed after diagnosis is similar in young patients [46].

Similar data we found in a univariated analysis study (with fasting C-peptide as depending variable) on a T1DM group, where we verified a positive correlation with the age of the patient at clinical diagnosis (r = 0.270; p = 0.001) and negative with the disease duration (r = -0.652; p < 0.001) and with the HbA1c value (r = -0.176; p = 0.029) of our population. So it was demonstrated that, apart from patients' age, disease duration as the exposure to hyperglycemia time are also important to the maintenance of residual C-peptide secretion.

Another widely discussed aspect in literature is the ability of pancreatic beta cells to regenerate. In a recent case report, where it was possible to assess this factor in a pancreas sample from an 89-year-old patient, T1DM (GADA, IA2 positive and lower fasting C-peptide) of recent diagnosis, submitted to surgery to neo pancreatic duct. Duplicating beta cells were detected in the pancreatic tissue through immunohistochemical analysis and potassium channel indicators [47]. Such case study shows that a potential pancreatic beta cell regeneration is a possibility to be considered while we are discussing the residual C-peptide secretion in T1DM.

The number of anti-islet auto-antibodies during the pre-diabetic stage and at diagnosis of T1DM shown a inverse relation with the residual beta cell [48]. This is easily verified when we compare T1DM in children and LADA [49].

At T1DM diagnosis, apart from the autoimmune insult, beta cells are being submitted to hyperglycemia itself by the glycotoxic effect, which also cooperates to decrease C-peptide secretion. This last effect could be demonstrated when a residual insulin secretion was compared in two groups of T1DM with the same clinical features during the first two years from clinical diagnosis, one submitted to intensive insulin therapy and other under conventional therapy [50]. In this study, it was observed that even through both groups present the same residual C-peptide secretion at diagnosis and after the 1^st ^follow-up year, by the end of the 2^nd ^year it was significantly higher in the intensive therapy group. However, it has been discussed if such effect was obtained through the removal of glycotoxicity or through the insulin immunomodulate effect.

Regarding immunomodulators, we evaluated nicotinamide in a double blind study during the 1^st ^year of T1DM diagnosis. In this study although the fasting C-peptide did not change, we did not see any differences between patients who used this vitamin and placebo [51].

Since a few years ago, some studies have been conducted using as immunomodulators the antigens involved in the autoimmune process against beta cells.

One of the first these clinical trials was a double-blind study, stage II which used a heat-shock protein peptide (Diapep27) [52]. In this study recently diagnosed T1DM patients received this peptide at their clinical diagnosis, and 1 to 6 months later. It was demonstrated that the group which received this vaccine presented significantly higher residual C-peptide secretion from the 7^th ^month and this result was until the end of the study (10^th ^month), while in the placebo group, there was a progressive reduction in the fasting-C peptide since the 1^st ^month of diagnosis.

Another study in this way used monoclonal anti-CD3 [53] antibody, which was also able to cooperate to C-peptide preservation during the 1^st ^year of diagnosis.

The most recent study for the denominated "secondary prevention of T1DM" used vaccine with GAD-65[54]. In this study, recently diagnosed T1DM patients received 20 μg GAD-alum on the 1^st ^and 30^th ^days of the protocol. It was demonstrated through this study, for patients who received this vaccine up to 6 months after clinical diagnosis, that the fasting C-peptide was significantly higher than the placebo group on the 10^th ^and 30^th ^month of the study.

The secondary prevention of T1DM has acquired increasing importance in the last few years due to the insufficiency of the latest large studies [54,55] for primary prevention and to the positive effect of the C-peptide residual secretion to prevent hypoglycemia[56] and the prevention of diabetic microangiopathy (nephropathy and neuropathy)[57]. These last actions should be related to a higher stability of glycemia in these individuals and to possible actions which would be intermediated through the insulin receptor or a specific C-peptide receptor. Such "hormone" would supposedly act through G protein, C-peptide receptor and protein kinase C (PKC) and MAPK and nuclear transcription factors [58]. Renal, circulatory and neural clinical actions have been related to the C-peptide [59].

## Conclusion

In short, T1DM is a progressive autoimmune disease mediated by T cells with destruction of beta cells. Up to now, we do not have precise methods to assess the beta cell mass, "*in vivo*" or "*ex-vivo*". The studies about its genetic susceptibility show strong association with class II antigens of the HLA system (particularly DQ). Other associations are weaker and depend on the studied population. A combination of precipitating events (virus, food factors and others) must occur at the beginning of the disease, and the intestinal barrier must play an important role in this process. There is a silent loss (immuno-mediated) of the beta cells mass which velocity has an inverse relation with the age, but it is influenced by genetic and metabolic factors. However, in the clinical diagnosis of the disease, there is a significant reserve of functioning beta cells. The goal of secondary prevention, by the immunomodulation of the process, is the preservation of such functional beta-cell reserve. It may be assessed through C-peptide values, which are important for glycemia stability and for the prevention of chronic complications in this disease.

However, the determinant risk factors of T1ADM, the autoimmune response initiators, the mechanisms which regulate the process toward beta cell failure and the factors which determine the time of clinical diabetes arrive are not fully known yet.

## Abbreviations

T1DM: type 1 diabetes mellitus; T1ADM: type 1A diabetes mellitus; LADA: latent autoimmune diabetes in adults; IPEX: immune dysfunction, polyendocrinopathy, enteropathy X-linked; T-regs: regulatory T cells; APECED: autoimmune polyendocrinopathy-candidiasis-ectodermal dystrophy; AIRE: autoimmune regulatory gene; GADA: glutamic acid decarboxilase antibodies; MHC: major complexo of histocompatibility; VNTR: variable number of tanden repeats; TEDDY: The Environmental Determinants of Diabetes in the Young; Th1: immune cellular response; Th2: immune humoral response; DAISY: Diabetes Autoimmunity Study in the Young; PTPN-22: protein tyrosine phosphotase non-receptor type 2; PKC: protein kinase C; MAPK: mitogen-activated protein kinase.

## Competing interests

The authors declare that they have no competing interests.

## Authors' contributions

SAD wrote and MBG reviewed this manuscript. Both authors read and approved the final manuscript
